# Animal Models for Crimean-Congo Hemorrhagic Fever Human Disease

**DOI:** 10.3390/v11070590

**Published:** 2019-06-28

**Authors:** Aura R. Garrison, Darci R. Smith, Joseph W. Golden

**Affiliations:** Virology Division, United States Army Medical Research Institute of Infectious Diseases, 1425 Porter Street, Fort Detrick, MD 21702, USA

**Keywords:** Crimean-Congo hemorrhagic fever virus, animal models, pathogenesis, medical countermeasures

## Abstract

Crimean-Congo hemorrhagic fever virus (CCHFV) is an important tick-borne human pathogen endemic throughout Asia, Africa and Europe. CCHFV is also an emerging virus, with recent outbreaks in Western Europe. CCHFV can infect a large number of wild and domesticated mammalian species and some avian species, however the virus does not cause severe disease in these animals, but can produce viremia. In humans, CCHFV infection can lead to a severe, life-threating disease characterized by hemodynamic instability, hepatic injury and neurological disorders, with a worldwide lethality rate of ~20–30%. The pathogenic mechanisms of CCHF are poorly understood, largely due to the dearth of animal models. However, several important animal models have been recently described, including novel murine models and a non-human primate model. In this review, we examine the current knowledge of CCHF-mediated pathogenesis and describe how animal models are helping elucidate the molecular and cellular determinants of disease. This information should serve as a reference for those interested in CCHFV animal models and their utility for evaluation of medical countermeasures (MCMs) and in the study of pathogenesis.

## 1. CCHFV as an Endemic and Emerging Pathogen

In 1973, Crimean-Congo hemorrhagic fever virus (CCHFV) was identified as the singular causative agent of two separate illnesses, Congo fever (identified in 1956) and Crimean fever (identified in 1944) [1,2]. CCHFV is a member of the *Nairoviridae* family in the order *Bunyavirales*, a group of enveloped tri-segmented negative stranded RNA viruses. Despite having been originally identified in West Central Africa and the Crimea, today the virus is endemic throughout a wide geographical area that includes Africa, Asia and Europe. The presence of the virus in these regions is directly correlated with the presence of the main arthropod vector of CCHFV, *Hyalomma spp* ticks [2,3]. While CCHFV is endemic in many areas, the expansion of the host-range of the ticks is allowing the virus to emerge in new areas [4]. In 2016, a fatal human case of CCHF was reported in Spain [5], six years after detection in the regional tick population [6]. The widespread endemic nature of CCHFV and the fact that it is emerging into new geographical regions led the World Health Organization (WHO) to declare it a priority pathogen.

CCHFV has a dichotomous relationship with animals and humans. While CCHFV infects a large number of wild and domesticated mammalian species, including bovines and ovines, and some avian species such as ostriches, the virus does not cause severe disease in these species [7]. Instead, infections in these animals are predominantly asymptomatic, often resulting in a viremia that can last >5 days [7,8] which helps maintain CCHFV in nature. In marked contrast, CCHFV infection in humans can lead to a severe, even life-threating, disease with key features that include coagulopathy, hepatic injury and neurological disorders [9,10]. An in-depth understanding of CCHFV-mediated pathogenesis has been hampered by the lack of animal models. However, several murine and non-human primate models have recently been developed which will provide a means to investigate CCHFV pathogenesis, in addition to providing a platform to bridge medical countermeasure (MCM) development to humans. Here, we review human CCHF disease in detail and describe how recent developments in animal models, in particular our own recent findings, can be used to better understand pathogenic mechanisms of CCHFV. Furthermore, we discuss the current development of MCMs and how animal models have been used to evaluate their therapeutic potential against CCHFV.

## 2. Virus Strain Genetic Diversity

CCHFV has a tripartite, negative-sense RNA genome comprising small (S), medium (M) and large (L) segments. The S segment encodes the nucleocapsid (N) protein, the M segment encodes the glycoprotein open reading frame (ORF) that is cleaved into two structural glycoproteins (G_N_ and G_C_) and nonstructural proteins, and the L segment encodes the RNA-dependent RNA polymerase (reviewed in [11,12]). CCHFV is the most genetically diverse arthropod-borne virus and nucleotide sequence differences between isolates can range from 20% for the S segments, 22% among the L segments, and up to 31% for the M segments [3]. Based on genetic differences, the CCHFV strains are divided into six to seven lineages depending on the RNA segments used and the labeling system [3,13,14,15,16]. The overall impact of genetic diversity on pathogenesis is poorly understood. Heterogeneity, along with other factors such as availability of advanced medical care and host factors may partially account for the broad global range in the case fatality rate (CFR) of 2–80% [1,17,18,19,20]. For example, the AP92 and AP92-like strains circulating in Greece and Turkey are associated with a low level of virulence and mortality despite evidence that there is an estimated 6% and 5.2% seropositivity, respectively, in the human population in these area [21,22,23]. In contrast, strains circulating in China have caused a high mortality rate of ~80% [24]. Strain genetic diversity needs to be considered in MCM development, especially for products targeting complex immunological epitopes, such as viral glycoproteins. Furthermore, understanding the mechanism(s) by which genetic factors impact virulence may help guide MCM design by identifying viral and host factors impacting disease outcomes.

## 3. Human Crimean-Congo Hemorrhagic Fever

### 3.1. Transmission

Humans are considered accidental hosts of CCHFV. Human infections result following bites from infected ticks or exposure to pulverized infected ticks feeding on agricultural animals (i.e., during sheep shearing) [2]. Another major route of human infection is exposure to the blood of infected wild or more commonly agricultural animals, which can be viremic but otherwise lack obvious signs of disease [2,7,25]. Human-to-human transmission through close contacts (i.e., family members) or nosocomial infections occurs with some frequency [9,10,26,27,28]. The risk of nosocomial infections is particularly high in situations where diagnosis of CCHF is unexpected or undetermined. This was recently demonstrated in Spain, where the primary CCHF patient was infected from a tick-bite and succumbed to the undiagnosed disease, however CCHFV was not suspected until a member of the medial care team presented with symptoms [5]. A similar scenario occurred following treatment of an American Soldier whose CCHF was not diagnosed until late during treatment, resulting in the infection of some of the medical caregivers [27]. Situations such as these may be increasing as CCHFV emerges in new areas.

### 3.2. Phases of Disease

CCHF occurs in four general phases: incubation, pre-hemorrhagic, hemorrhagic, and convalescence. Upon infection, the incubation phase typically lasts for about 3–7 days, and the timing probably depends on the route of exposure (i.e., tick-bites, respiratory exposure or needle sticks) and the viral dose [2,29]. Following incubation, there is a pre-hemorrhagic phase lasting from 1–7 days that manifests as a rapid onset of acute febrile illness with severe fever, headache, nausea, diarrhea, muscle aches, photophobia, and other non-specific prototypical “flu-like” symptoms [1,2,29]. Soon after onset of illness, circulating virus can be detected in blood by reverse transcriptase polymerase chain reaction (RT-PCR). Some patients progress to the hemorrhagic phase, which typically lasts from 1–3 days. Patients with high levels of circulating virus (e.g., 9 log_10_ genomes per ml of plasma) have a poorer prognosis that those with lower circulating virus levels, thus circulating virus levels may serve as one predictor for the progression to the hemorrhagic phase and disease outcome [30]. During this phase, viremia decreases and hemorrhages, ranging from petechiae to large areas of ecchymosis to profuse bleeding, are often more pronounced in CCHF than in other viral hemorrhagic diseases [9]. In severe cases, the coagulation cascade is disrupted and the patient rapidly progresses and succumbs to infection due to disseminated intravascular coagulation (DIC), bleeding, multi-organ failure, and shock [31,32]. Mortality rates of 2–80% have been reported for various CCHF outbreaks and it is not clear if the large range is due to differences in the virus itself, or to other factors such as route of exposure or dose of the infecting virus (reviewed in [11,33]). CCHF can be especially dangerous during pregnancy [1], this is likely a result of the immunocompromised status of the mother [34]. Fetal/neonatal mortality is very high and in at least one comprehensive analysis it approached 58.5% [35]. Maternal mortality was about 34%, which was considered higher compared to the overall lethality in humans. An additional concern with CCHFV infections during pregnancy is the enhanced risk of nosocomial infections. Interestingly, CCHF in children may be milder for reasons that are unclear [36,37].

### 3.3. Hematology and Coagulopathy

Thrombocytopenia is a common, almost universal, symptom of CCHF [31]. Leukocytosis or leukocytopenia is very common during disease and the former is part of the Swanepoel criteria for assessing disease severity [32]. Presence of thrombocytopenia (<150,000 platelets/mm^3^) and leukocytosis (>9000 lymphocytes/mm^3^)/leukocytopenia (<3000 lymphocytes/mm^3^) are useful indicators of probable cases of CCHF in many endemic areas. However, other studies did not observe a correlation between development of severe disease and leukocytosis. Coagulation abnormalities are a hallmark of CCHF and during the hemorrhagic phase can range from gingival bleeding to DIC. Elevations in prothrombin, activated thromboplastin, and thrombin times occur to varying degrees during CCHF with significant differences between mild/moderate and fatal cases. For example, an activated partial thrombin time (APPT) of >60 s and/or fibrinogen levels <110 mg/dL, as well as other laboratory indicators of DIC are predictors of a severe infection [9,38,39,40,41]. Other factors contributing to hemostatic instability may include effects on endothelial cells directly and indirectly due to viral replication. Endothelial cell activation may augment platelet aggregation [42].

### 3.4. Innate Immunity and Hyper-Inflammatory Cytokine Responses

While inflammatory cytokines and chemokines are essential for successful host responses against infectious agents, an overabundance of these molecules can contribute to pathological damage [43]. There is some correlation with the levels of the inflammatory cytokines interleukin (IL)-6, IL-8 and tumor necrosis factor (TNF)-α with fatal/severe CCHF [38,44,45]. In particular, higher levels of IL-6 and IL-8 by day 5 post-disease onset are strongly associated with fatal outcomes and can predict poor outcomes [44]. Furthermore, high levels of the monocyte chemokine MCP-1 (CCL2) also correlated with severe human disease [44]. Other studies have identified the presence of secreted trigger receptor expression on myeloid cells-1 (sTREM-1), which is an amplifier of inflammatory responses, in the serum of those with severe CCHF [46]. These epidemiological data suggest that the host inflammatory response(s) may play an important role in viral pathogenesis. However, definitive evidence that aberrant levels of these inflammatory factors drive pathogenic processes during CCHF remain to be experimentally demonstrated.

Genetic variation in human type I interferon (IFN-I) responses may partially influence CCHF severity. Retrospective analyses of CCHF human cases have identified polymorphisms in toll-like receptors (TLR), TLR8/9 and TLR3 are reported to play a role in acute disease [17,18]. These epidemiological data suggest a model whereby some TLR polymorphisms limit IFN-I activation in response to CCHFV allowing for enhanced viral replication that in turn enhances disease severity. In general, genetic variation in pathogen sensing systems, such as the TLR networks, can influence host susceptibility to viral infection [47].

### 3.5. Adaptive Immune Response

Human IgM and IgG antibody responses can be detected against the glycoproteins (GP38, G_N_ and G_C_), and nucleocapsid (N) protein in CCHF survivors [48,49,50]. This includes development of neutralizing antibodies responses for which G_C_ is the only known target [12,51]. Antibody responses are generally undetectable in fatal cases of CCHF and even in those who develop severe disease responses can be of low titer which may precipitously increase in potency in the weeks/months following infection [8]. Antibody responses can be maintained long term, suggesting life-long protection against CCHFV [52,53]. Using a novel gamma interferon enzyme-linked immune absorbent spot (ELISpot) test consisting of peptides derived from the N protein and the G_C_ glycoprotein, Goedhals, et al. reported the existence of memory CD8^+^ T-cell responses in ten of eleven survivors several years subsequent to virus exposure [54]. No immunodominant epitope was identified and most responses targeted peptides from N protein, however at least two survivors had CD8^+^ T-cell responses against G_C_. Thus, long-lived CD8^+^ T-cell responses targeting CCHFV are produced during infection and present in survivors. Although some studies indicate that antibody (convalescent human plasma) can protect against severe disease [55], the immune correlates required for protection against CCHFV are not clear and the relative importance of humoral and cytotoxic T-cell responses for protection of humans against primary and secondary virus exposure remain to be determined experimental.

### 3.6. Organ Specific Pathogenesis

#### 3.6.1. Liver Pathogenesis

CCHF human disease is often associated with mild to severe liver injury, including fulminant hepatic failure [5,27,32,56,57]. In fact, clinical characterization of CCHF severity is partially based on elevated liver enzymes with severe disease determined by the Ergonul criteria as aspartate aminotransferase (AST) values of ≥700 U/L or alanine aminotransferase (ALT) values ≥900 U/L [56] or by the Swanepoel criteria which includes AST values of ≥200 U/L or ALT values of ≥150 U/L [32]. The normal ranges for these enzymes in healthy humans are 10–40 U/L and 7–56 U/L, respectively. Histological analysis of CCHFV-liver infection has been reported in a limited number of autopsy studies [5,57,58]. These findings generally reveal CCHFV antigen in hepatocytes and non-parenchymal liver cells such as Kupffer cells and liver endothelial cells. Liver injury is characterized by Kupffer cell hyperplasia and hepatocellular necrosis. While the evidence is far from clear, it has been suggested that CCHFV replication within hepatocytes, and their resultant destruction, directly leads to organ dysfunction [57]. This has been supported experimentally by in vitro data showing that CCHFV causes ER-stress and apoptosis in the Huh7 hepatocyte-like cell line [59]. However, the mechanistic details of liver cell loss during CCHFV infection are poorly understood. Hepatocytes are not only critical for detoxification, amino acid/protein metabolism and copper homeostasis, but also produce many of the serum proteins, including coagulation factors II, VII, IX, X, fibrinogen and plasminogen, in addition to the carrier proteins such as albumin and transferrin [60]. Hepatocytes and sinusoidal endothelial cells produce the glycoprotein hormone thrombopoietin, which is involved in production and differentiation of megakaryocytes and ultimately controls serum platelet levels [60]. Thus, liver injury associated with CCHF may further erode hemodynamic stability due to limiting the availability of clotting factors and the restoration of platelet levels. How the CCHFV liver insult contributes holistically to CCHF is also not well characterized, but clearly hepatic injury is central to severe disease in many cases.

#### 3.6.2. Neuropathogenesis

CCHFV can be neurotropic in humans and neuropsychiatric disorders occurring during CCHF have been reported [1,9]. In some studies, neurological disorders were the primary symptom [27,61,62,63,64]. The neurological disorders induced by CCHF include mood alteration, confusion, disorientation, aggression, cerebral/cerebellar edema and encephalopathy. Subdural hematoma resulting in cerebral hemorrhage is considered rare, but have been reported [61]. Cerebral/cerebellar edema can result in cerebellar tonsil herniation and lead to fatal outcomes [27]. One study suggests that the brain is largely not affected by CCHFV [65]. However, that study included a small patient sample size and none of the patients developed severe disease. Hepatic injury may also contribute to neurological impairment and one case reported the development of hepatic encephalopathy [66].

#### 3.6.3. Cardiac and Respiratory Disease

Both cardiac and pulmonary sequelae can occur during CCHF [67,68,69,70]. Respiratory distress includes the development of acute respiratory disease syndrome (ARDS) with parenchymal and alveolar infiltration, pleural effusion, coughing that includes hemoptysis, dyspnea and pulmonary hemorrhage. Additionally, a retrospective study identified the presence of pulmonary artery enlargement, indicative of pulmonary hypertension, in several CCHF patients [71]. ARDS-like symptoms are closely associated with acute inflammatory responses and may manifest as a result of endothelial infection and the vascular damage caused by CCHFV. Some studies suggest chest x-ray or thorax computer tomography may be useful in detecting and responding to respiratory symptoms during CCHF [72,73]. In addition to lung involvement, some reports indicate CCHF also impacts the heart [69]. Severe cases of CCHF resulted in lower left ventricular ejection and pericardial effusion. Moreover, cardiac congestion and interstitial edema were also reported. Because only a few studies have evaluated the cardiac and respiratory disease consequence of CCHF, more work is needed to fully understand how these factors influence outcomes during infection.

## 4. Small Animal Models

### 4.1. History of Murine Model Development

CCHFV does not cause disease in immunocompetent adult rodents, including mice, rats, guinea pigs and hamsters [8,25,74]. Until 2010, the only available models were neonatal mice and neonatal rats which were first used in 1967 by Chumakov and colleagues [2,75]. However, Bereczky, S. et al. discovered that strain 129 mice lacking the type I interferon receptor A (IFNAR^−/−^) were susceptible to CCHFV and produced a lethal/severe disease model [76]. Subsequently, these studies were repeated in C57BL/6 mice also lacking the type I interferon (IFN-I) receptor [77]. Additionally, CCHFV produces severe disease in STAT-1^−/−^ mice and mice lacking both the IFN-I receptor and IFN-gamma receptor (IFNAGR^−/−^). These animals have deficiencies in both IFN-I and type II interferon (IFN-γ) signaling [78,79]. We recently developed a novel murine system by exploiting an antibody against IFN-I receptor A (MAR1-5A3) that was previously shown to produce severe disease models with other unrelated viruses [80,81]. This antibody produces a transient IFN-I blockade in mice and results in consistent lethal/severe CCHFV infection [82,83]. The advantage to this model is it creates the same phenotype as an IFN-I receptor knockout animal in virtually any wild-type or transgenic mouse without the need for cross-breeding. The disease produced in the antibody-mediated IFN-I blockade model is essentially identical to the disease observed in genetic KO animals with similar mean times to death. In addition to conventional mouse systems, Spengler et al. developed a novel humanized mouse model by transferring human CD34^+^ stem cells into NOD-SCID-gamma (NSG)-SGM3 mice, which are extremely immunodeficient mice lacking mature T-cells, B-cells, and natural killer (NK) cells and have defects in cytokine signaling due to lack of the common gamma chain. Infection of these mice with CCHFV produces neurological disease [84]. Below we describe how these murine systems are being used to evaluated CCHF pathogenic processes in addition to MCM development. Table 1 is a list of key CCHFV animal models reported in the literature to date. Table 2 lists strains of nairoviruses commonly used in animal models.

### 4.2. Routes of Infection

The natural routes of CCHFV infection in humans are respiratory and through tick-bites (subcutaneous) (see above). In mice lacking IFN-I signaling, severe disease will ensue subsequent to infection via the intranasal (IN), subcutaneous (SC), intramuscular (IM) and intraperitoneal (IP) routes [77,78,83]. In IFNAR^−/−^ mice, IN infection is the least efficient and requires a higher viral challenge dose [77]. The mean time to death (MTD) in IFN-I deficient mice is generally between 5–8 days, depending on virus dose and route of infection. In C57BL/6 mice with IFN-I blockade infected via IP and SC routes, severe disease will occur, however, lethality is reduced in the latter route (60%) [99]. In side by side comparisons, the MTD is similar to transgenic mice [82]. Lethality in the humanized mice is much more delayed at ~18 days following IP exposure [84]. Overall, these findings indicate that infection in mice can be facilitated by natural routes of CCHFV exposure and provides opportunities to study the molecular and cellular requirements for CCHFV progression across natural barriers.

### 4.3. Hematology and Coagulopathy

Similar to humans, mice develop hematological and coagulation abnormalities including lymphocyte depletion from the spleen, activated partial thromboplastin times, increased plasma fibrinogen and decreased platelets [77,78]. In some animals, fibrin deposition can be detected in the livers which is indicative of DIC [83].

### 4.4. Hyper-Inflammatory Cytokine Response

Consistent with human CCHF, there is a marked increase in inflammatory cytokines (IL-6, IL-8 and TNF-α) and chemokines (CCL2, CXCL1 and CCL5) following CCHFV infection in IFN-I deficient mice (genetic or by antibody blockade) [77,78,83]. Serum cytokine and chemokine levels increase precipitously for the duration of disease reaching peak levels at the time the animals meet euthanasia criteria. TNF-α protein also increases in the liver of infected animals [83]. Furthermore, transcripts for TNF-α, IL-1a, IL-1b, CCL2, CCL5, CXCL1, in addition to other chemokines, significantly increase in the livers of infected mice 4 days post virus exposure. These increases are accompanied by significant increases in transcripts for several IFN-I response factors such as Ddx58/RIG-I, MYD88 and Ifih1/MDA-1 [83].

### 4.5. Adaptive Immune Response

There is a limited understanding of the adaptive immune components required for protection against primary and secondary CCHFV challenge. Wild-type mice develop antibody responses against N, G_N_, G_C_ and GP38 [48,51,82]. This includes neutralizing antibodies [51]. While IFN-I signaling clearly protects mice from severe disease, perhaps unsurprisingly innate immunity alone is not sufficient for protection [83]. We infected C57BL/6 or Rag2-deficient mice with CCHFV and after 15 days both groups of mice did not lose weight. However, when IFN-I was blocked on day 15 by antibody-treatment, Rag2-deficent mice rapidly succumbed to disease and were euthanized, but in contrast BL6 mice were protected. This experiment was repeated using mice lacking CD8+ T-cells or B-cells (µMT^−/−^). Both groups were protected when IFN-I was blocked on day 15 suggesting that CD8+ T-cells and B-cells redundantly contribute to protection from primary challenge [99]. This work is very preliminary and more studies are needed to identify the adaptive immune components critical for protection against primary infection. Only a few studies have investigated the requirements for protection against secondary challenge. One group reported that vaccine-produced antibody alone does not protect mice against challenge subsequent to passive transfer, suggesting that antibodies are not critical for vaccine-mediated protection [100]. Recently, we found that a non-neutralizing antibody targeting GP38 can protect against lethal infection in adult mice (see below), however several neutralizing antibodies failed to provide protection or delay MTD [48]. This antibody also protected when given after virus exposure. Therefore, antibody may be a key component for protection against CCHFV. For future work, the IFN-I blockade model may be useful by allowing protective efficacy to be evaluated in mice lacking critical arms of the adaptive immune system. The Rag2-deficient murine model [83] may also provide insight through conventional adaptive transfer studies using antigen naïve or experienced T-cell and B-cells.

### 4.6. Organ Specific Pathogenesis

#### 4.6.1. Liver Pathogenesis

CCHFV is a hepatotropic virus in humans and this is recapitulated in murine systems. The liver is the major target during infection of neonatal mice and viral antigen can be found in Kupffer cells and hepatocytes [85]. In adult mice with IFN-signaling defects, viral protein and viral RNA is present in many tissues including brain, heart, lung, pancreas and kidney, but overall the liver is the primary target of replication and the primary, if not exclusive, site of pathology [77,78,83]. However, there are some impacts on the spleen including lymphocytosis and diffuse neutrophil infiltration. In mice, serological evidence of liver damage includes marked increases in the liver enzymes AST/ALT starting 2–3 days post-infection, these levels continue to rise throughout the disease time course. Histopathologic changes in the liver are a characteristic of CCHFV infection in humans [57] and include Kupffer cell hypertrophy, hepatocellular degeneration/necrosis and occasional fibrin thrombi. Liver pathological changes develop in mice infected with multiple strains of virus, including the prototypical laboratory strain IbAr 10200 [77,82] and a human isolate called Afg09-2990 [83]. Kupffer cells are targeted during CCHFV infection in humans [57] and this is reproduced in the murine models. In mice, viral RNA and antigen (N) can be initially detected in Kupffer cells, subsequently spreading to other liver cells as infection proceeds, a sequence that also appears to occur in humans [101]. Curiously, while severe disease does not ensue in mice with intact IFN-I systems, we observed Kupffer cell infection in Rag2-deficient mice when IFN-I signaling was still intact. Thus, Kupffer cells may play an important role in viral dissemination to and initial replication within the liver. Kupffer cells (CLEC4^+^) are rapidly lost 24 h prior to animals succumbing to disease [83]. A similar loss of Kupffer cells in human livers has not been investigated.

Now that it is clear CCHFV reliably produces hepatic injury and severe disease in mice, this platform can be used to continue to explore the mechanism(s) underlying this process. In our initial studies into the mechanism behind liver pathology, we found that mice infected with CCHFV, with IFN-I signaling blocked by antibody, had infiltration of CD45+ cells into the liver and this coincided with increases in immune cell transcripts for NK cells, CD8^+^ T-cells (CTLs), neutrophils and dendritic cells [83]. Because cytotoxic immune cells targeting virally-infected cells might facilitate liver cell destruction, we explored their role in CCHFV-mediated liver injury. In mice lacking T-cells, B-cells and NK cells (NSG and Rag2-deficient mice), liver injury occurs with kinetics similar to wild-type mice and identical pathological features. In fact, chronic hepatitis develops in Rag2-deficient mice even in the presence of active IFN-I signaling [83]. Note that this hepatits occurs in the absence of outward signs of disease such as weight loss. These data indicated that cytotoxic immune cells are likely not key drivers of hepatic injury. Using Nanostring and ELISA analysis, multiple studies have shown large increases in TNF death receptor pathways in the serum and livers of CCHFV infected mice [77,83]. This is consistent with models supported in the literature that argue CCHF may be in part an inflammatory disorder, with the presence of aberrant levels of cytokines contributing to the overall organ dysfunction resulting in severe disease [38,44]. Indeed, the liver is particularly vulnerable to TNF-α, FasL and TRAIL and other studies have shown these molecules alone, independent of infection, can result in liver injury [102,103,104,105,106]. There was no correlation between the presence of viral RNA and liver cell apoptosis, suggesting that bystander cell death, perhaps from death receptor pathways, was occurring in the liver. These findings support a model whereby the inflammatory response itself is important for driving the pathogenic process in infected mouse livers. Further studies are needed to determine if inflammatory cytokines are critical in the development of hepatic injury in infected mice.

#### 4.6.2. Neuropathogenesis

A model for CCHF neurological disease was recently developed. This model capitalizes on humanized mice, which are NSG-SGM3 mice injected with human CD34^+^ stem cells [84]. Human CD34+ cells are hematopoietic stem cells that upon transfer to the mouse develop into multi-lineage human immune cells, as a result these mice also express human IL-3, granulocyte-macrophage colony-stimulating factor (GM-CSF) and human steel factor (SF). Infection of these mice with CCHFV strain Turkey-200406546 (Turkey-2004), but not from strain Oman-199809166 (Oman) (Table 2 and Table 3), produces a lethal disease with mice meeting euthanasia criteria between 13 and 23 days. Viral RNA is present in various tissues, with the highest levels in the spleen, liver and brain. Some mild liver injury was detected in at least one animal. However, histopathological changes are most severe in the brain and are accompanied by CCHFV-positive immunostaining. Pathological changes included signs of meningoencephalitis and astrocyte gliosis. In mice with severe disease, edema and vascular congestion were also observed. Focal areas of virus positive cells could be detected in the habenular nuclei and hypothalamus. This is particularly interesting as CCHF has been observed in the hypothalamus of infected humans [62]. This model will be critical in addressing the cellular and molecular factors driving CCHFV neurological disease.

#### 4.6.3. Cardiac and Respiratory Disease

In IFNAR^−/−^ or STAT-1^−/−^, virus can be detected in the heart and lungs [77,78]. However, in these animals the dominating pathological responses after IP or SC challenge occurred in the liver and spleen. There were no signs of pathology in the heart or lungs in either study. More work is needed to elucidate the impact of CCHFV in the heart and lung and determine if a pathology similar to that which occurs in humans can be produced. For these studies, infection of mice via the IN route may be useful. However, it is possible that mice succumb to disease too rapidly for pathological responses to develop in the heart and lung.

## 5. Tick-Transmission Models

Ticks represent not only the vector for CCHFV transmission, but it is thought that they are the sole reservoir for viral maintenance in nature [2,115]. Ticks in the genus *Hyalomma* are the main vector of CCHFV where it is maintained in vertical and horizontal transmission cycles [116]. CCHFV transmission by infected ticks is an important mode of human and animal infection. Accordingly, animal models emulating tick–host transmission are invaluable in understanding this aspect of CCHFV biology. These studies are inherently difficult due to complexities of tick propagation and maintenance in the laboratory. Additionally, handling CCHFV requires a high-containment laboratory (BSL4) adding another layer of difficulty. Historically, several studies have successfully reproduced tick to tick, vertebrate to tick, and tick to vertebrate transmission of CCHFV in a laboratory setting [2,116,117,118]. This included a study by Levi and Vasilenko demonstrating that CCHFV could infect *Hyalomma plumbeum* ticks subsequent to feeding on rabbits injected with CCHFV [116,117]. Ticks (larval, nymphs and adults) feeding on infected rabbits were positive for CCHFV thus confirming vertebrate to tick CCHFV transmission. CCHFV transmission from small animal to tick to large animals was shown by Shepard et al. Here, ticks fed on CCHFV-infected rabbits could subsequently transmit CCHFV to sheep [8]. Although the virus briefly replicated in sheep these animals did not develop disease, rather the sheep seroconverted. Curiously, one study found that naturally infected ticks could transmit CCHFV to guinea pigs, and contrary to other studies the animals developed severe disease with some mortality [119]. However, ticks artificially infected with virus did not produce disease in guinea pigs [120], possibly indicating natural infection of ticks allows the virus to be transmitted in a way that facilitates disease. These historic studies were performed outside the now requisite high containment facilities needed to work with CCHFV. More recently, Gargili, et al. developed models for working with ticks in high containment [118]. Using *Hyalomma marginatum* ticks, this group developed models for propagating uninfected ticks using guinea pigs, mice and rabbits. Rabbits proved to be the ideal host for tick growth. Furthermore, using STAT-1 KO mice infected with CCHFV strain IbAr 10200, the group was able to demonstrate successful transmission of virus to ticks feeding on infected animals as determined by RT-PCR. A method of virus inactivation in ticks handled in the BSL-4 environment was also reported, and involved submerging infected ticks in 100% ethanol for 1.5 h followed by formalin and bleach for 2 h. This foundational work presents an opportunity for more in-depth evaluation of CCHFV-tick biology. An important experiment using the currently available models would be one that could reliably demonstrate tick transmission to IFN-I deficient mice to confirm the development of severe disease via a natural route of infection. This model would allow for the evaluation of the primary sites of CCHFV replication in the dermis where ticks feed and transmit virus. Viral replication in dendritic and Langerhans cells in the dermal barrier may be important for viral replication [121]. If a tick transmission mouse model could be developed, it would allow exploration of the mechanism(s) required for tick transmission of CCHFV to humans, including the role of dendritic cells in the dermal barrier.

## 6. Non-Human Primate Models

The development of an NHP model that recapitulates human CCHF disease has been a challenging area of research. Earlier studies of CCHFV infection of African green monkeys, baboons, and patas monkeys were unsuccessful [2,25,122]. Recently, a cynomolgus macaque severe disease model was described that establishes the first immunocompetent animal model for CCHF [88]. NHPs were infected with the European human clinical isolate of CCHFV, strain Kosova Hoti, using an intravenous (IV) or combined IV and subcutaneous (SC) high dose (5 log_10_ TCID_50_) exposure. The animals became viremic and developed a severe and sometimes fatal disease characterized by inflammatory immune responses, elevated liver enzymes, increased clotting times, thrombocytopenia, leukopenia and fever, which are all representative of human cases of CCHF. Histopathology demonstrated that CCHFV mainly targeted the liver and spleen where in situ hybridization identified viral RNA in the hepatocytes, Kupffer cells, and endothelial cells.

Our group recently expanded upon the cynomolgus macaque model by comparing the pathogenesis of an Asian human clinical isolate, CCHFV strain Afg09-2990, to that of strain Hoti (Smith, D., et al. manuscript under review [89]. These two strains were injected IV into two separate groups of cynomolgus macaques and the disease course was monitored. Regardless of strain, all animals demonstrated signs of clinical illness, viremia, significant changes in clinical chemistry and hematology values, and serum cytokine profiles consistent with CCHF disease in humans. In contrast to the earlier study, no animals met euthanasia criteria and all NHPs fully recovered. In contrast, 75% (3/4) of the animals that were exposed IV with CCHFV strain Kosova Hoti in the earlier study met euthanasia criteria [88]. More animals in that study experienced signs of severe disease such as body/facial edema and bleeding. Differences in scoring criteria between both studies may account for why no animals met euthanasia criteria in the current study. Other possible explanations could include variables in virus stock, dose, and genetic background of NHPs. These differences should be examined further in an effort to refine and standardize the CCHF cynomolgus macaque model.

While mortality may not be as constant in the CCHFV NHP model, fever seems to be a universal feature. The use of temperature monitoring by telemetry in our study did offer a significant advantage over single point temperature monitoring to determine when animals became febrile. We detected a febrile response that lasted on average between 5 and 7 days post-infection, whereas animals infected in the earlier study detected elevated temperatures only on day 1 post-infection in two of four animals [88]. The detection of a lower febrile response in the earlier study is likely due to measuring temperature changes rectally in anesthetized animals. The use of telemetry-based temperature monitoring offers a more sensitive and accurate means to evaluate the febrile response, which is an important endpoint criterion for evaluating the effectiveness of MCMs. For example, NHP models aimed at satisfying the FDA animal rule for MCMs against Venezuelan equine encephalitis virus (VEEV), rely on telemetry for fever monitoring [123,124]. Aside from fever monitoring, the CCHFV NHP model offers other criteria to evaluated disease, including platelet levels, liver enzymes and viremia. These parameters should provide clear criteria to monitor any candidate MCMs.

Interestingly, our study provided insight into the ability for CCHFV to persist and potentially cause long-term sequela following infection. Three male monkeys that survived infection had evidence of unilateral inflammation in the testis where both CCHFV antigen and RNA were detected in one of these animals. This study provides the first direct evidence that CCHFV can replicate and persist in the male genital tract, which has important implications for human sexual transmission. Unexpectedly, the histopathology also revealed that six additional animals had granulomas and/or granulomatous lesions in the lungs, tracheobronchial lymph node, and/or liver that were suspect for mycobacterial infection. This finding allowed us a unique opportunity to observe disease parameters of both pathogens in the same host. Tuberculosis threatens millions of lives worldwide and is the leading cause of death due to a bacterial pathogen. Concurrent mycobacterial infection with other infectious diseases has been described, but not for CCHFV despite the geographic overlap of these two pathogens. Interestingly, CCHFV antigen and RNA were detected within the granulomas of two of these tuberculosis positive animals. This study is the first to demonstrate the persistence of CCHFV in the testes of NHPs and in animals concurrently affected by latent tuberculosis, which has important public health implications for these emerging pathogens.

The development of the cynomolgus macaque model represents an important advancement in the field where an immunocompetent CCHF animal model is now available to study pathogenic disease mechanisms and evaluate candidate medical countermeasures. Adding further value is the ability to use two genetically unrelated strains, Hoti and Afg09-2990, which both produce disease in the NHP. This model should be further refined to determine reproducibility by evaluating variables such as virus strain/stock, dose, and genetic background of NHPs. Furthermore, the mechanism and impact of viral RNA persistence on the development of long-term sequela is an important area of future research in the NHP model.

## 7. Countermeasure Development

### 7.1. MCMs Use in Humans

Despite CCHFV having been identified as the causative agent of human CCHF >40 years ago, very few drugs or vaccines have been tested in humans. There are currently no FDA licensed products to treat or prevent CCHF. The only vaccine product evaluated in humans is a inactivated whole virus vaccine produced in mouse brain; the virus is inactivated by chloroform and heated at 58 °C and adsorbed on Al(OH)3 (Table 3). The protective efficacy of this vaccine has not been established, but it is given to at risk individuals (butchers/animal slaughter workers) in Bulgaria [114,125]. Two products, the nucleoside-analog ribavirin and hyperimmune human plasmas/serum, produced from either vaccinated persons or CCHFV survivors, have been used as post-exposure therapeutics (Table 4). Ribavirin is currently the most extensively deployed post-exposure antiviral [27,126,127,128,129,130,131,132]. However, the protective efficacy of ribavirin against CCHFV is not clear; some studies suggest that ribavirin does not protect against mortality but other studies indicate its therapeutic potential. Retrospective pooled analysis indicates that ribavirin reduces mortality by 44% compared to no treatment [133]. Several of the studies assessing the effectiveness of ribavirin also examined the combined therapy of ribavirin and a corticosteroid. In these studies, the addition of a corticosteroid was demonstrated to be beneficial in patients with severe disease [128,132,133,134]. The efficacy of hyperimmune sera or plasma has also been mixed with some studies showing protection and others failing to do so [55,135,136]. Overall, the lack of MCMs for CCHFV is related to the historic lack of animal models, the limited number of human CCHF cases, and the economic limitations of drug marketability for rarer infectious diseases.

### 7.2. Evaluation of MCMs in Animal Models

Mouse infection models have been the exclusive animal system used in the evaluation of CCHFV MCMs. This includes the initial evaluation of ribavirin in neonatal mice in 1993 [85]. Extensive reviews on CCHFV-targeting MCMs exist elsewhere for vaccines and therapeutics [135,141,142,143]. A list of current major MCMs being developed against CCHFV is shown in Table 3 (vaccines) and Table 4 (therapeutics). Interestingly, of the products that have been used in humans, including the inactivated Bulgarian vaccine and immunotherapeutics, only ribavirin has been evaluated in an animal model. Here, we focus on the strengths and weaknesses of various murine systems in the evaluation of MCM efficacy.

#### 7.2.1. Vaccines

**Figure 1:**
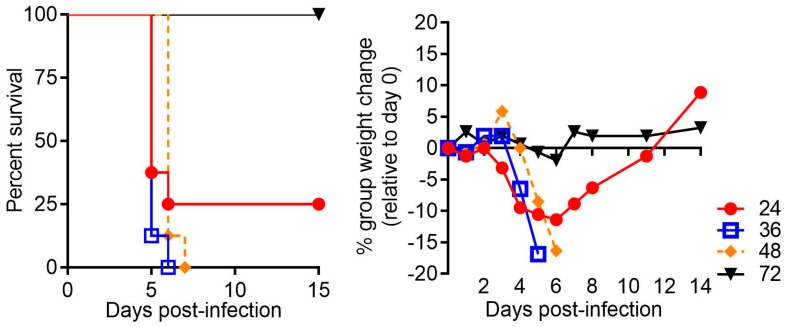
Lethal Crimean-Congo hemorrhagic fever virus (CCHFV) infection in mice treated with MAb-5A3 post-challenge. C57BL/6 mice(*n* = 8/group) were infected with 100 plaque forming units/mL CCHFV strain Afg09-2990 by the intraperitoneal route as described in [104] and at the indicated times post-infection (24 h, 36 h, 48 h or 72 h) were treated with MAb-5A3 (2.5 mg) which disrupts type I interferon signaling. Survival and group weights were monitored for 15 days and plotted using Prism software.

Protective efficacy of various CCHFV vaccines including DNA, MVA-vectored and adenovirus-vector vaccines have been reported in IFNAR^−/−^ mice, including those on the 129 and C57BL/6 backgrounds [82,107,112,113]. Another group reported protective efficacy of a vaccine expressing N on a Bovine herpesvirus type 4 vector in mice lacking both type I and type II interferon receptors [79]. Rodriguez, et al. reported the protective efficacy of a recombinant a vesicular stomatitis virus (rVSV) expressing the glycoproteins in STAT-1^−/−^ mice from lethal challenge [86]. In contrast, vaccination of STAT-1^−/−^ mice with a glycoprotein subunit vaccine produced neutralizing anti-CCHFV glycoprotein antibody responses, but mice were not protected against challenge [111]. These differences may be due the stains used with the former study using strain Turkey-2004 [86] and the study that did not see vaccine-induced protection using strain IbAr 10200 [111]. Therefore, caution must be exercised as the STAT-1^−/−^ murine model may not be conducive to vaccine study due to hypersensitivity to infection and/or poor anamnestic responses, which may hinder the study of some potentially protective vaccines, especially subunit vaccines. IFN-I signaling is important for the generation of antigen-presenting cell maturation, driving T cell and subsequent B cell responses, as well as promoting the generation of memory T and B cells pools [144]. Because IFN-I signaling can be critical for vaccine immune responses, we developed the antibody-mediated IFN-I blockade CCHFV severe disease model by templating studies reported by Sheehan et al. for other viruses [80]. This system allows the dynamic control of IFN-I signaling, thus permitting vaccination in an immune intact animal before exposure to virus and lessens the impact IFN-I disruption may have on secondary immune responses following challenge. Indeed, IFN-I blockade can be initiated up to 48 h post-exposure with CCHFV strain IbAr 10200 or Afg09-2990 and mice still develop severe disease (Figure 1).

#### 7.2.2. Immunotherapeutics

We recently screened several murine neutralizing and non-neutralizing antibody monoclonal antibodies shown to protect neonatal mice from lethal infection [51] and found only one protected adult mice [48]. This antibody, mAb-13G8, is non-neutralizing and targets GP38 (Table 1). Utilizing the antibody-mediated IFN-I blockade model in mice lacking functional Fc-receptors or complement activity due to a C3-deficiency, we found that mAb-13G8 protection requires complement activity. Thus, and perhaps unsurprisingly, neonatal mice are not entirely reliable for assessing the protective efficacy of antibody-based MCMs. Interesting, neutralizing antibodies failed to confer protection or reduce the MTD in the murine model, suggesting that CCHFV might spread within the host via cells such as macrophages or extracelluar vesicles.

#### 7.2.3. Small Molecule Inhibitors

In neonatal mice, ribavirin reduced lethality and delayed the mean time to death [85]. However, protection in adult STAT-1^−/−^ and IFNAR^−/−^ mice is limited [78,87,140]. The observed limited protection of ribavirin in mice harmonizes with the, albeit limited, human data [27,126,127,128,129,130,131,132]. Giving the limitations of ribavirin at protection, the search more efficacious small molecule inhibitors against CCHFV is underway. Studies in adult mice have identified favipiravir (T-705) as a promising candidate [87,140]. Favipiravir protects mice against lethality by strains Afg09-2990, IbAr 10200 and Kosova Hoti (Hoti) even when administrated post-infection. The broad-spectrum fusion inhibitor arbidol did not protect in the mouse model against strain Afg09-2990 [140].

### 7.3. The Impact of Strain Heterogeneity on MCM Protection

CCHFV strains are genetically diverse with upward of seven distinct clades [145] and consequentially there exists some antigenic heterogeneity strains [146,147]. Accordingly, the ability to evaluate the protective efficacy of any MCM against diverse strains of CCHFV is critical, in particular vaccines and immunotherapeutics. For example, we were able to determine that mAb-13G8 produced against strain IbAr 10200, does not protect mice well from the heterologous strain Afg09-2990, likely due to variability in the GP38 molecule [48]. The added dynamic of having multiple strains of CCHFV to assess MCM protective efficacy is highly advantageous and for mouse models, this includes use of strains IbAr 10200, Hoti, Afg09-2990 and strain Turkey-2004 (Table 3). These strains produce a lethal disease in mice lacking IFN-I signaling.

### 7.4. Down-Selection of CCHFV Murine Models for MCM Evaluation

Overall, the adult murine models are proving useful in early MCM development for vaccines, immunotherapeutics and small molecule inhibitors against CCHFV. However, there is little standardization regarding models used by different groups. We propose that future studies focus on using the IFNAR^−/−^ mice, either the BL6 or 129 background, as this model has the fewest genetic mutations and maintains consistent susceptibility to several CCHFV strains. These mice are also readily available from commercial sources. Additionally, the use of the IFN-I antibody blockade may be powerful the evaluation of MCM protection, particularly vaccines. Finally, the advent of a viable NHP model should greatly assist in bridging laboratory discoveries into candidate human-safe MCMs against CCHFV.

## 8. BSL2 and BSL3 Surrogate Models

Because CCHFV research requires BSL4 containment and many researchers do not have access to such facilities, several groups have developed surrogate nairovirus murine models. Hazara virus (HAZV) is a nairovirus isolated from the *Ixodes redikorzevi* tick and is a member of the CCHFV serogroup [93] (Table 5). Evidence to date indicates that HAZV is non-pathogenic in humans and can be manipulated in BSL2 environments. Dowall, et al. demonstrated that similar to CCHFV, HAZV is pathogenic in IFNAR^−/−^ mice [148] (Table 3 and Table 5). HAZV infection in IFNAR^−/−^ mice led to severe disease with a MTD of ~5 days depending on viral dose. Histopathological changes in the liver and spleen were detected and are analogous to that of CCHFV infection of mice. Recently a novel nairovirus called Tolfa virus (TFLV) was isolated from *Haemaphysalis flava* ticks and *Haemaphysalis fomsensis* ticks in Japan. TFLV is also in the CCHFV serogroup. Shimada, et al. found that this virus, though considered non-pathogenic in humans, produced severe disease in IFNAR^−/−^ (A129 background) mice [95]. Infection in these mice resulted in pathological effects in the intestinal tract and was lethal with a MTD of ~4–5 days. Liver involvement in TFLV murine infection was not specified in the published reports.

In addition to HAZV and TFLV, another nairovirus termed Dugbe virus (DUGV) has shown promise as a CCHFV surrogate. DUGV is a member of the Nairobi sheep disease virus serogroup [152,153]. Infection of mice either immunocompromised by cyclophosphamide treatment (within 48 h) or IFNAR^−/−^ mice results in a lethal disease which included respiratory tract involvement (lung edema) and also a neurological disease [150,151]. Contrary to HAZV and TFLV, DUGV has been reported to occasionally cause human disease, particularly in children [101]. For this reason, study of DUGV requires BSL3 containment. Interestingly, one report suggested that a human isolate of DUGV (IbH11480), contrary to tick-isolates, could produce disease in immunocompetent mice [98]. Despite DUGV not being in the same serogroup as HAZV and TFLV, the possibility that tick and human isolates have differing phenotypes in immunocompetent mice may allow for important insight into viral genetic factors influencing nairovirus pathogenesis. Overall, the use of BSL2 and BSL3 surrogates for CCHFV is promising and suggest that these viruses, in particular HAZV, should continue to be investigated as surrogate models for CCHFV pathogenesis.

## 9. Conclusions

The geographical distribution of CCHFV and ticks capable of supporting CCHFV propagation in nature is expanding into new areas [4]. Most recently, *Hyalomma rufipes*, a tick species which can support virus propagation in nature, were identified in England [154]. Thus, there is an urgent need for not only rapid diagnostics to identify CCHF cases [155,156], but also MCMs that can mitigate disease, particularly in a post-exposure setting. The advent of new models for studying disease in rodents and NHPs lays the foundation for important advancements for CCHFV research. These systems will be critical in elucidating the complex host-pathogen dynamics leading to CCHFV-induced organ injury and severe disease. Furthermore, the NHP model in particular will greatly aid in MCM design and development. Hopefully within the next few years, effective products can be brought into advanced development that can protect at risk populations against CCHFV.

## Figures and Tables

**Table 1:** CCHFV animal models.

Animal Model	Virus Strain(s)	Virus Dose	Route(s) of Infection	% lethality	Time to Death [days]	Salient Features	REF
Neonatal mice	IbAr 10200	100 Lethal-dose units	IP	100	3 d	Do not predict immunotherapeutic protection behavior in adult rodents, Ribavirin protects against lethality	[85]
STAT-1^−/−^ mice	IbAr 10200, Turkey-2004	10-1000 PFU	SC, IP	20–100	3–6 d	hepatic injury, subunit vaccines may not protect well in this model. 10 PFUtick dose is only 20% lethal, higher doses uniformly lethal	[78,86]
IFNAR^−/−^ mice	IbAr 10200, Afg09-2990, Hoti	10-10,000 TCID_50_ or PFU	SC, IP, IN, IM	>90	4–8 d	Prototypical rodent model for CCHFV, C57BL/6 or 129 background develop severe disease. Strain Hoti has a reduced MTD	[76,77,82,87]
IFNAR^−/−^, IFNAGR^−/−^ mice	Ank-2	100 TCID50	IP	100	4–6 d	Used to evaluate N subunit vaccines	[79]
C57BL/6, BALB/c, B6:129	IbAr 10200, Afg09-2990, Hoti	100 PFU	SC, IP	>90 (IFN-I blockade)	5 d	No disease ensues unless IFN-I signaling is blocked by antibody (MAR1-5A3)	[48,83]
Rag2^−/−^ mice	Afg09-2990, Hoti	100 PFU	IP	100	4–5 d after disruption of IFN-I signaling	Hepatitis in mice with active IFN-I signaling, disruption of IFN-I signaling results in 100% lethality similar to normal mice	[83]
SGM3 Humanized mice	Turkey-2004, Oman-199809166	1 × 10^4^ TCID_50_	IP	0 or 100	15–23 d	Neurological disease ensues absent of systemic (visceral) disease. Only strain Turkey produced severe disease and lethality. Oman is not lethal	[84]
Cynomolgus Macaques	Hoti, Afg09-2990	5log_10_ TCID_50_ and 1 × 10^6^ PFU	IV, SC and IV/SC combo	0–60	6–7 d	Disease model with fever, increased liver enzymes, thrombocytopenia, leukocytopenia. In some studies animals meet euthanasia criteria	[88,89]

**Table 2:** Strains of nairoviruses used in animal systems.

Virus	Strain	Origin	Passage History	Animal Model	REF
**CCHFV**	IbAr 10200	1966, tick-isolate (*Hyalomma excavatum*), Nigeria	9× SMB ^1^, 3× HepG2 ^2^	Mice	[90]
	Afg09-2990	2009, human-isolate, fatal case, Afghanistan	3× Vero ^2^, 2× Huh7 ^2^	Mice, NHP	[91]
	Kosova Hoti	2001, human isolate, fatal case, Kosovo	2× VeroE6 ^2^	Mice, NHP	[92]
	Oman-199809166	1998, human-isolate, outcome unknown, Oman	2× VeroE6, 1× SW13 ^2^	Mice	[84]
	Turkey-200406546	2004, human-isolate, outcome unknown, Turkey	1× SMB, 1× SW13	Mice	[84]
	Ank-2	2012, human-isolate, outcome unknown, Turkey	3× SW13	Mice	[79]
**HAZV**	JC280	1964, tick-isolate (*Ixodes redikorzevi*), Pakistan	SMB	Mice	[93,94]
**TOFV**	Tok-Hfla-2013	2013, tick-isolate (*H. formosensis*), Japan	Not passaged, homogenized ticks used	Mice	[95]
**DUGV**	IbAr 1792	1964, tick-isolate (*Amblyomma variegatum*), Nigeria	SMB	Mice	[96]
	KT281/75	1975, tick-isolate (*Amblyomma variegatum*), Nigeria	5× BSC-1 ^2^, 2× SMB	Mice	[97]
	IbH11480	1966, human-isolate, Nigeria	5× SMB	Mice	[98]

^1^ Suckling mouse brain (SMB). ^2^ Cell lines (HepG2, Huh7, Vero E6, SW13, BSC-1).

**Table 3:** CCHFV vaccine MCMs evaluated in humans and laboratory animals.

Vaccine.	Treatment Regimen	Route of Vaccination	Animal Species/Strain	% Protection	Target(s)	Mechanism of Protection	Human Efficacy Data	REF
MVA-GP	1 × 10^7^ PFU/dose, 2 doses	IM	IFNAR^−/−^(A129)	100	M-segment glycoproteins	antibody appeared irrelevant	N	[107]
M-segment DNA vaccine	25 µg DNA, three doses	IM electroporation	IFNAR^−/−^(C57BL/6), or C57BL/6 (mAb 5A3 treated upon challenge)	60–70	M-segment glycoproteins	neutralizing and total antibody titers do not correlate with protection	N	[82]
rVSV expressing M-segment ORF	1 or 2 doses of 10^7^ PFU/dose	IP	STAT-1	100	M-segment glycoproteins	antibody against glycoproteins, and neutralizing antibody titers but mechanism is unclear	N	[86]
G_N/_G_C_ and N DNA vaccine and/or VLPs	50 µg DNA; 1 × 10^6^ VLPs, three doses varying combinations	intradermal electroporation (DNA), IP (VLP)	IFNAR^−/−^(A129)	100	G_N_, G_C_ and N	unknown	N	[108]
Bovine Herpesvirus N subunit vaccine	100 TCID_50_, two doses	IM	IFNAGR^−/−^	100	N	unknown	N	[79]
CCHF virus-like replicon particle with M-segment	1 dose of 10^5^ TCID50 or 10^3^ TCID50	SC	IFNAR^−/−^	10^3^ TCID50 (80%), 10^5^ TCID50 (100%)	M-segment glycoproteins	unknown	N	[109]
MVA-NP	1 or 2 doses of 10^7^ PFU/dose	IM	IFNAR^−/−^(A129)	0	N	not protective	N	[110]
G_N_ ectodomain or G_C_ ectodomain subunit vaccines	2 doses 7.5 µg G_C_ or 15 µg G_N_	IP	STAT-1	0 ^+^	G_N_ or G_C_	not protective	N	[111]
Formalin inactivated cell culture derived CCHFV mixed with alum	3 doses of 5, 20, or 40 µg	IP	IFNAR^−/−^	5 µg dose (60%), 20 and 40 µg (80%)	Whole virus	antibody against glycoproteins, and neutralizing antibody titers but mechanism is unclear	N	[112]
Adenovirus N subunit vaccine	1.25 × 10^7^ IFU	IM	IFNAR^−/−^(C57BL/6)	33–78	N	prime/boost more protective	N	[113]
Mouse brain-derived chloroform and heat inactivated CCHFV strain V42/81 ^#^ adsorbed on Al(OH)_3_	1 mL doses (day 0 and 30, 1 y and every 5 y thereafter (given March-April)	SC	humans	Unknown	Whole virus	antibody against glycoproteins and N, and T-cell response to N but mechanism is unclear	Y	[114]

^#^ Human data; ^+^ Either subunit

**Table 4:** CCHFV therapeutic MCMs evaluated in humans and laboratory animals.

Class	MCM	Treatment Regimen	Route of Delivery	Animal Species/Strain	Post-Exposure Protection	% Protection	Target(s)	Mechanism of Protection	Human Efficacy Data	REF
**Immunotherapeutic**	CCHF-bulin ^#^	3–9 mL, 1–5 d or longer	IM	humans	Y	>60(human)	antibody targets unidentified	human convalescent plasma	Y	[55]
	CCHF-venin^#^	30 mL combined with 30 mL of CCHF-Bulin	IV	humans	Y	100(human)	antibody targets unidentified	human convalescent plasma	Y	[55]
	mAb-13G8	1 mg/dose, two doses	SC, IP	IFNAR^−/−^, mAb 5A3 treated C57BL/6 mice	Y	70–100	GP38	may involve complement	N	[48]
**Small-molecule**	Ribavirin ^#^	500 mg (oral), 30 mg/kg–7.5 mg/kg IV	oral, SC ^#^, IV ^#^, IP ^^^	humans, mice (STAT-1 and IFNAR^−/−)^	Y	20–80 Mice Unclear * (Human)	Nucleoside-analog	targets viral RNA synthesis	Y *	[78,85,87,131,137,138,139]
	Favipiravir	300 mg/kg	IP	IFNAR^−/−^mice	Y	100	Nucleoside-analog	targets viral RNA synthesis	N	[87,140]

^#^ Human data; ^ Route in mice; * Conflicting human efficacy data

**Table 5:** BSL2 and BSL3 nairovirus mouse models.

Virus	Animal Model	Virus Strain(s)	Virus Dose	Route(s) of Infection	% Lethality	Time to Death [days]	Salient Features	REF
**HAZV**	Neonatal mice	JC280	10^3^–10^4^ LD_50_	IC	100	2.5–3 d	neuronal destruction, viremia and high titers in liver	[149]
	IFNAR^−/−^ mice (A129)	JC280	40000, 1000 and 10 PFU	ID	70–100	4–7 d	liver damage, histopathological changes in spleen and lymph nodes	[148]
**TOFV**	IFNAR^−/−^ mice (A129)	Tok-Hfla-2013	10^-3^–10^3^ FFU	SC	0, 25 or 100	3–6 d	gastrointestinal disorder, 10^−3^ FFU dose not lethal, 10^−2^ FFU 25% lethality, higher doses are uniformly lethal	[95]
**DUGV**	Neonatal mice	KT281/75	0.3-1522 PFU	IN	100	3–6 d	highest titers in brain	[150]
	CD-1 mice + cyclophosphamide	KT281/75	>4.2 × 10^4^ PFU	SC, IN	0–80	< 40 d	respiratory and neurological disease ensues in cyclophosphamide treated mice but only after IN challenge. SC challenge is not lethal	[150]
	IFNAR^−/−^ mice (A129)	IbAr 1792	100-1000 PFU	IC, IP	100	2–5 d	Neurological disease	[151]
	CD-1 mice	Ib11480	2488 PFU	IN	Not specified	Not specified	neurological disease, did not require immunosuppression	[98]
